# Temperature-controlled porcine eye holder for observing intraocular temperature during cataract surgery

**DOI:** 10.1038/s41598-023-31070-4

**Published:** 2023-03-15

**Authors:** Keiichiro Minami, Saori Yaguchi, Hiroko Bissen-Miyajima

**Affiliations:** Department of Ophthalmology, Tokyo Dental College Suidobashi Hospital, 2-9-18 Kandamisaki-Cho, Chiyoda-Ku, Tokyo, 101-0061 Japan

**Keywords:** Lens diseases, Experimental models of disease

## Abstract

During cataract surgery, the intraocular temperature changes when irrigating low-temperature fluid and ophthalmic viscosurgical devices (OVDs) are inserted in the anterior chamber, and such a temperature variation affects the unfolding of the intraocular lens (IOL). A porcine eye holder was developed for simulating temperature conditions in clinical surgery by maintaining the ocular temperature close to the body temperature. An aluminum holder was designed to fit porcine eyes and maintain the ocular temperature at approximately 36 °C, while surgery was performed at a room temperature of 20 °C. Intraocular temperature was monitored using a thermocouple sensor placed close to the posterior capsule in the vitreous cavity. Temperatures and microscopic image of the anterior chamber were simultaneously recorded. With the use of the eye holder system, the intraocular temperature unstable during surgery was observed, and there were significant reductions during hydrodissection, irrigation and aspiration, OVD insertion in the capsule, and OVD removal after IOL implantation.

## Introduction

Enucleated porcine eyes have been used in the ex vivo evaluation of the performance and effects of phacoemulsification and aspiration (PEA)^1,2^, femtosecond laser-assisted cataract surgery^3,4^, ophthalmic viscosurgical devices (OVDs)^5–7^, and implantation of intraocular lens (IOL)^8,9^. Porcine eyes are usually preserved at low temperatures, whereas balanced salt solution (BSS) and OVDs used in the ex vivo experiments are stored at lower temperatures in the surgery room or laboratory, such as 20–25 °C or 68–77 degrees Fahrenheit. Hence, the temperature of the porcine eyes during the experiments is lower than that of the live eyes, which is closer to the body temperature. As viscoelastic behavior depends on temperature, this temperature difference could affect the ex vivo evaluation in cataract surgery. The unfolding time of the hydrophobic acrylic IOLs is shortened by increasing the ambient temperature^10,11^, whereas prompt IOL unfolding is important when using toric IOLs to avoid spontaneous rotation occurring shortly after implantation^12,13^. However, to our knowledge, the temperature difference between ex vivo and clinical conditions has not been addressed in cataract surgery. Hence, we developed an eye holder that brought the ocular temperature to the body temperature and measured the intraocular temperature during cataract surgery.

## Results

**Figure 1:**
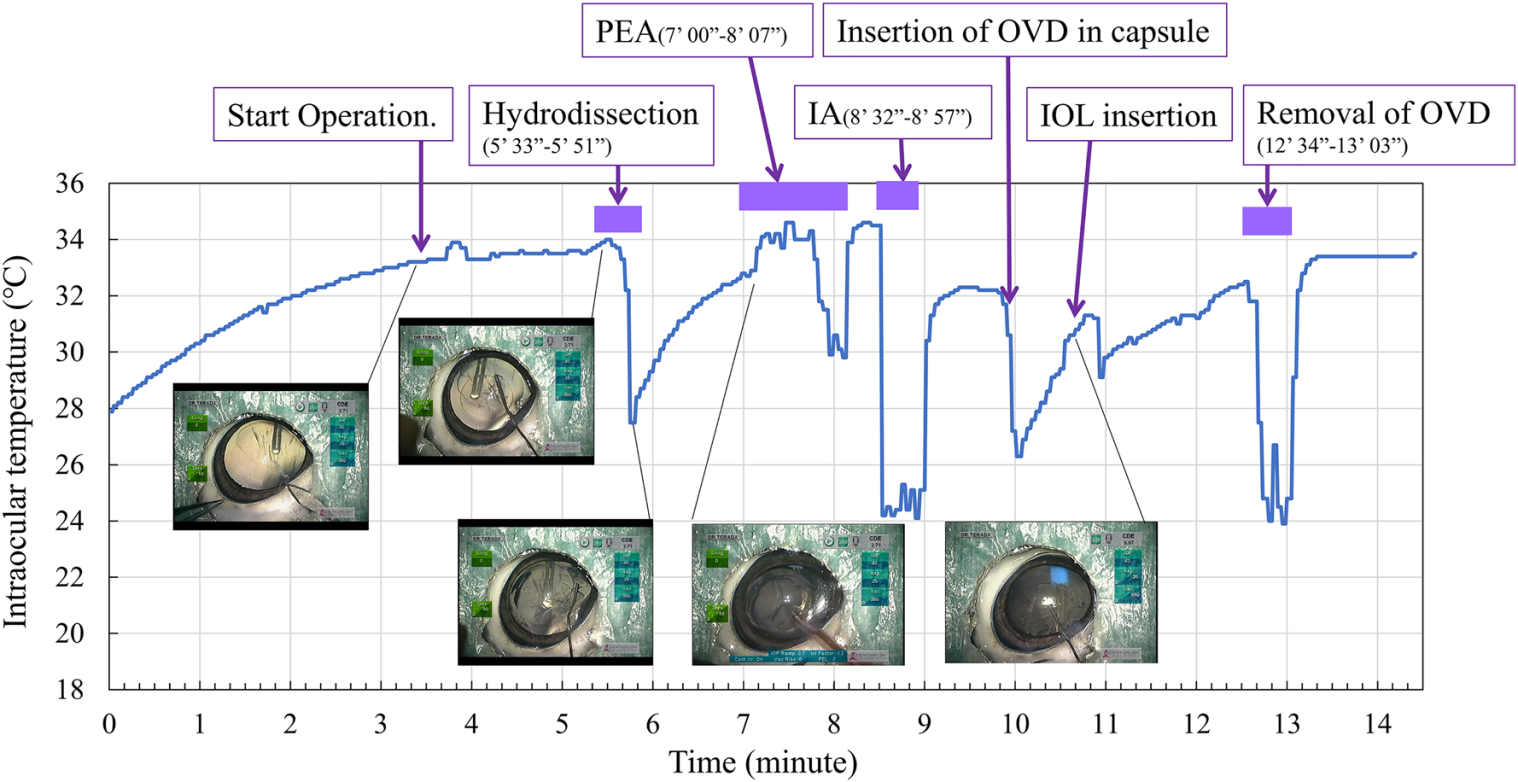
Intraocular temperature during cataract surgery in a typical case (Case #1 in Table 1). The series of procedures and time durations are indicated with the corresponding boxes and bars. The microscopic surgery images of procedures have been presented in the lower portion of the image. PEA: phacoemulsification and aspiration, I/A: irrigation and aspiration, OVD: ophthalmic viscosurgical device, IOL: intraocular lens.

Figure 1 shows the time-course of the changes in the intraocular temperature observed in a typical case (Case #1 in Table 1) together with the microscopic surgery images. Surgery was started when the intraocular temperature was stable (at approximately 3 min 25 s). In the microscopic surgery images, a thermocouple sensor was found in the vitreous cavity. The temperature was stable during the insertion of the OVD into the anterior chamber and continuous curvilinear capsulorrhexis (CCC). During hydrodissection, the intraocular temperature decreased by 6.5 °C when the inserted BSS reached the posterior capsule. After the procedure, the temperature gradually increased to 32.9 °C. After a slight decrease at the end of PEA, there was a steep decrease by 10 °C at the beginning of irrigation and aspiration (I/A), followed by a quick recovery by 6 °C at the end of the procedure. During insertion of the OVD in the capsule, the temperature decreased by 5 °C, but recovered gradually. No obvious decrease was observed during IOL insertion. During the removal of the OVD, the temperature drastically changed, as observed in the I/A procedure.

**Table 1:** Reduction in the intraocular temperature during four surgical procedures performed at a room temperature of 20 °C and eye-holder temperature setting of 36 °C.

Case No	Hydrodissection	I/A	Insertion of OVD in the capsule	Removal of OVD
1	34.0 to 27.5 °C (6.5 °C)	34.5 to 24.2 °C (10.3 °C)	32.2 to 26.3 °C (5.9 °C)	32.5 to 24.0 °C (8.5 °C)
2	34.3 to 21.4 °C (12.9 °C)	28.9 to 21.9 °C (7.0 °C)	27.8 to 23.9 °C (3.9 °C)	32.5 to 22.4 °C (10.1 °C)
3	34.8 to 19.8 °C (15.0 °C)	29.4 to 24.7 °C (4.7 °C)	29.5 to 28.3 °C (1.2 °C)	32.9 to 23.2 °C (9.7 °C)
4	34.6 to 23.1 °C (11.5 °C)	31.6 to 30.1 °C (1.5 °C)	30.8 to 27.3 °C (3.5 °C)	34.9 to 23.8 °C (11.1 °C)

Data are presented as maximum to minimum (difference).

I/A, irrigation and aspiration; OVD, ophthalmic viscosurgical device.

Notable temperature changes were observed during the hydrodissection, I/A, insertion of the OVD in the capsule, and removal of the OVD procedures. Table 1 shows the changes in the intraocular temperature during these procedures observed in four porcine eyes. During hydrodissection, the intraocular temperature changed from approximately 34 °C to 19.8–27.5 °C, which indicated that the insertion of BSS into the posterior capsule decreased the intraocular temperature by 6.5–15.0 °C. One the other hand, the changes were not consistent during the I/A and OVD insertion procedures. During OVD removal, the temperatures decreased from the range of 32.5–34.9 °C to the range of 22.4–24.0 °C.

## Discussion

We developed a temperature-controlled porcine eye holder and measured intraocular temperature during cataract surgery. This first experiments revealed that the intraocular temperature decreased during hydrodissection, I/A, OVD insertion in the capsule, and OVD removal. Previous measurements before membrane peeling in 36 human eyes showed that the mean intraocular temperature behind the crystalline lens and in the middle of the vitreous cavity was 32.4 °C (standard deviation [SD]: 1.41) and 33.8 °C (SD: 0.95), respectively^14^, which was consistent with the current results before hydrodissection. It was demonstrated that the eye holder would simulate a temperature condition similar to that of live eyes as well as changes in the intraocular temperature during cataract surgery in patients.

No significant temperature change was observed when the low-temperature fluid was inserted into the anterior chamber, except during hydrodissection, I/A, OVD insertion in the capsule, and OVD removal. When the OVD was filled in the anterior chamber before hydrodissection, the crystalline lens was placed anteriorly to prevent direct contact between the OVD and vitreous cavity. During PEA and IOL implantation, the capsule was still filled with the previously inserted OVD; hence, the vitreous cavity was not affected by the insertion of the low-temperature BSS into the anterior chamber. We speculated that the presence of a crystalline lens or warm fluid could provide insulation from the low-temperature fluid in the anterior chamber. Further investigations are warranted to confirm this.

Either rapid or gradual recovery of the intraocular temperature was observed after the temperature reduction. Rapid increase in the intraocular temperature was observed after I/A and OVD removal, while gradual increase was observed after hydrodissection and OVD insertion in the capsule. Rapid changes were observed when there was no or a slight amount of OVD in the anterior chamber. Before PEA, the anterior chamber was filled with the OVD, and most of them was aspirated during PEA. Before OVD removal, the OVD was in the capsule, but it was diluted during IOL implantation. In contrast, gradual increase in the intraocular temperature was observed in the presence of elastic materials such as a crystalline lens and OVD in the anterior chamber. The heat capacity of an elastic material is higher than that of a liquid such as BSS; therefore, it takes more time for the temperature to increase. The heat capacity of the BSS and OVD should be measured to confirm this.

The current measurements resulted that the use of low-temperature BSS and OVD induced a decrease in the intraocular temperature. It is anticipated that the temperature decrease could be reduced further by using warmed BSS or OVD. Warmed OVD effectively shortens the unfolding time after IOL insertion^10,11^. Because it is difficult to increase the temperature of the surgical room, it would be more practical to warm the OVD before surgery. Utilizing a tube heating system within an aspiration line would be effective in warming the BSS to the desired temperature. Further considerations are required to control the temperatures of the BSS or OVD in use.

There are some limitations. First, there are fundamental differences in temperature changes between porcine eyes in the holder and live human eyes. Temperatures of a human eye is maintained by the arteries in in the choroid coat and abundant arteries are placed in the uvea. On contrast, the porcine eye in the holder is maintained from the sclera, so that recovery to the body temperature in live human eye would be faster. Size of eye ball would influence: larger size of porcine eye require longer time to change intraocular temperature. While it is hard to compare such differences, the current setup would be useful to stimulate and understand the temperature condition during surgery. Next, the number of eyes examined was insufficient, and research using enough porcine eyes is required. Moreover, the holder-preserved BSS flowed out from the eyes, causing the pooled BSS in the tray to disturb the temperature control. The temperature on the side face of the aluminum holder was measured and maintained at approximately 36 °C, but it occasionally decreased to 27–30 °C when the BSS flowed out from the eyes. The eye holder system should be improved by adding drains to the tray. Lastly, we could not investigate the effect of the decrease in the intraocular temperature on the IOL unfolding because of the iris. Porcine eyes without the iris^4^ are needed to observe the IOL behavior in the capsule.

## Methods

### Temperature-controlled porcine eye holder

**Figure 2:**
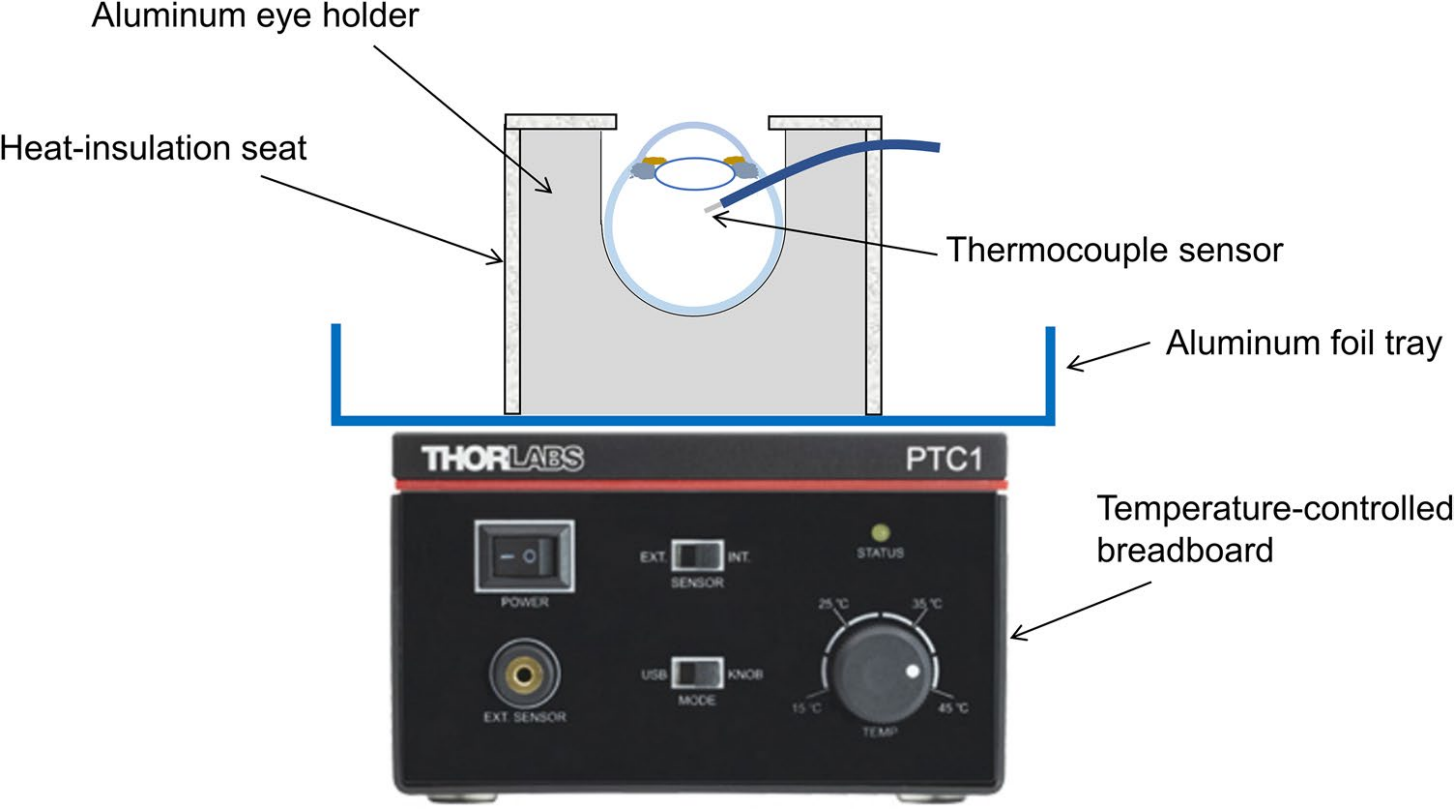
Schematic representation of the temperature-controlled eye holder.

As temperatures of a human eye is maintained by the arteries in the choroid coat, our eye holder is designed to maintain temperatures of the entire sclera. An aluminum eye holder with a semispherical hole on the top was designed for the placement of the eye. It was placed on a temperature-controlled breadboard (PTC1/M, Thorlabs, Newton, NJ, USA) with an interposing tray of aluminum foil between the holder and breadboard for reserving the fluid used in surgery (Fig. 2). The breadboard was equipped with thermoelectric coolers with a maximum heat load of 18 W to regulate the temperature in increments of 0.1 °C, and the breadboard temperature was set at 36 °C. The side faces of the aluminum holder were covered with a heat-insulation seat to minimize the influence of low ambient temperatures. On the top face, there was a heat-insulation seat for opening the cornea.

Four porcine eyes enucleated within 5 h were obtained from a local slaughterhouse (Tokyo Metropolitan Central Wholesale Market, Tokyo, Japan). Each eye was placed in the eye-holder and OVD was inserted into gaps between an eye and the eye-holder. A thermocouple sensor was inserted into the vitreous cavity of the porcine eye to measure the intraocular temperature. The sensor tip was placed close to the crystalline lens posterior for avoiding direct contact with the lens capsule^14^. Intraocular temperature was recorded using a temperature logger (TC0309, Perfect Prime, London, UK) at 1-s intervals. It was intended to place the tip of the sensor inside the vitreous cavity behind the crystalline lens to avoid direct contact with the lens capsule^14^.

### Cataract surgery

The cataract surgery was performed on the porcine eyes for stimulating intraocular temperature during cataract surgery. The surgical experiments were carried out using enucleated porcine eyes (commercially available) and not on live human and animal eyes.

After confirming that the intraocular temperature was stabilized close to the body temperature, the surgery was started. Both the intraocular temperature and video images of the microscopic surgery were recorded. Through a clear corneal incision, conventional cataract surgery was performed by one surgeon (S.Y.) using CENTURION® system (Alcon Laboratory, Fort Worth, TX, USA). PEA settings were an aspiration rate of 35 mL/min, vacuum limit of 380 mm Hg, and ultrasonic oscillation setting of 30%. As the BSS and OVD had been preserved for a couple of days in the surgical room, it was assumed that their temperatures would be the same as the room temperature (approximately 20 °C). The surgical procedures comprised OVD insertion into the anterior chamber, CCC, hydrodissection, coaxial PEA, I/A, insertion of the OVD in the capsule, insertion of a hydrophobic acrylic IOL (SN60WF, 25.5 D, Alcon Laboratory) using a Monarch injector, and removal of the OVD.

### Analysis

Changes in the intraocular temperature during each surgical procedure were examined by referring to the video and temperature records. If obvious changes in terms of reduction were found during certain procedures, the decreased temperatures were measured.

## Data Availability

The datasets generated during the current study are available from the corresponding author on reasonable request.
